# Triglyceride–glucose index is independently associated with left ventricular outflow tract obstruction in hypertrophic cardiomyopathy

**DOI:** 10.17305/bb.2026.14428

**Published:** 2026-07-01

**Authors:** Tolga Kunak, Özkan Kayhan, Ayşegül Ülgen Kunak

**Affiliations:** 1 Department of Cardiology, Akdeniz University Faculty of Medicine, Antalya, Türkiye; 2 Department of Cardiology, Kepez State Hospital, Antalya, Türkiye; 3 Department of Cardiology, University of Health Sciences, Antalya Training and Research Hospital, Antalya, Türkiye

**Keywords:** Triglyceride–glucose index, insulin resistance, hypertrophic cardiomyopathy, LVOT obstruction, echocardiography

## Abstract

Insulin resistance and metabolic dysregulation are increasingly recognized as contributors to myocardial remodeling and phenotypic heterogeneity in hypertrophic cardiomyopathy (HCM). While the triglyceride-glucose (TyG) index offers a simple surrogate marker of insulin resistance, its relationship with left ventricular outflow tract (LVOT) obstruction in HCM patients remains underexplored. This study aimed to investigate the association between the TyG index and LVOT obstruction in a retrospective observational cohort of 124 adult HCM patients. LVOT obstruction was defined as a resting or provoked gradient of ≥30 mmHg. Clinical, laboratory, and echocardiographic data were systematically evaluated. The TyG index was computed from fasting triglyceride and glucose levels. Multivariable logistic regression analysis, with the TyG index scaled per 0.1-unit increment, was used to assess independent associations. Receiver operating characteristic (ROC) curve analysis determined discriminatory performance. Patients with obstructive HCM exhibited significantly higher TyG index values compared to those without obstruction (9.07 ± 0.25 vs. 8.76 ± 0.21, *P <* 0.001). The TyG index maintained an independent association with LVOT obstruction after adjusting for relevant clinical and echocardiographic covariates (per 0.1-unit increase: OR 1.84, 95% CI 1.40–2.42, *P <* 0.001). Furthermore, the TyG index demonstrated moderate correlations with both resting (r = 0.519, *P <* 0.001) and provoked (r = 0.557, *P <* 0.001) LVOT gradients. ROC analysis indicated good discriminatory performance (AUC: 0.826, 95% CI: 0.752–0.901), with a cut-off value of 8.75 yielding 87.9% sensitivity and 47.0% specificity. In conclusion, the TyG index was independently associated with LVOT obstruction in HCM patients, with higher values observed in those with the obstructive phenotype. This suggests a potential link between metabolic status and disease manifestation in HCM. Given its cost-effectiveness and accessibility, the TyG index may offer valuable complementary clinical insights. However, this association requires cautious interpretation, and prospective studies are essential to establish its clinical and prognostic utility.

## Introduction

Hypertrophic cardiomyopathy (HCM), the most prevalent inherited cardiac disease, affects approximately 1 in 500 individuals in the general population [1]. Characterized by unexplained left ventricular hypertrophy (LVH), HCM manifests with diverse clinical phenotypes, including both obstructive and non-obstructive forms [2]. Left ventricular outflow tract (LVOT) obstruction, defined by a resting or provoked gradient ≥30 mmHg, occurs in about one-third of patients and is associated with more severe symptoms, progression to heart failure, and an increased risk of adverse cardiovascular outcomes [3, 4]. Current American Heart Association/American College of Cardiology (AHA/ACC) guidelines emphasize the assessment of both resting and provoked LVOT gradients due to their central role in symptom burden, risk stratification, and therapeutic decision-making in HCM patients [5]. Consequently, early identification of patients at risk for obstruction is of significant clinical importance.

Metabolic alterations are increasingly implicated in the pathophysiology of cardiac hypertrophy and remodeling. Insulin resistance, in particular, is a known contributor to LVH, diastolic dysfunction, and adverse cardiovascular outcomes [6]. The triglyceride–glucose (TyG) index, a validated surrogate marker of insulin resistance, has been linked to arterial stiffness, coronary artery disease, and incident diabetes [7–9]. Recent studies have also demonstrated associations between a higher TyG index and LVH in both hypertensive and metabolic syndrome populations [10, 11].

Despite growing evidence supporting the TyG index as a cardiovascular risk marker, its relationship with phenotypic heterogeneity in HCM remains poorly understood. Specifically, whether metabolic dysregulation contributes to the obstructive phenotype has not been clearly established. Given the emerging recognition of metabolic and inflammatory pathways in myocardial remodeling, evaluating simple surrogate markers of insulin resistance may provide additional insight into disease expression in HCM.

Therefore, this retrospective observational study aimed to investigate the association between the TyG index and LVOT obstruction in patients with HCM. We additionally evaluated the discriminatory performance of the TyG index for identifying obstructive physiology and explored its relationship with echocardiographic markers of structural remodeling.

## Materials and methods

### Study population

This retrospective observational study included consecutive adult patients (≥18 years) diagnosed with HCM between January 2018 and July 2025. Medical records, laboratory data, and echocardiographic examinations were reviewed from institutional databases. A total of 168 patients were initially screened. Forty-four patients were excluded due to incomplete laboratory data (*n* ═ 18), incomplete echocardiographic assessment (*n* ═ 10), prior septal reduction therapy (*n* ═ 6), advanced chronic kidney disease (estimated glomerular filtration rate [eGFR] <30 mL/min/1.73 m^2^) (*n* ═ 5), or active systemic inflammatory or infectious disease (*n* ═ 5). The final study population comprised 124 patients. The patient selection process is summarized in Figure 1.

**Figure 1.**
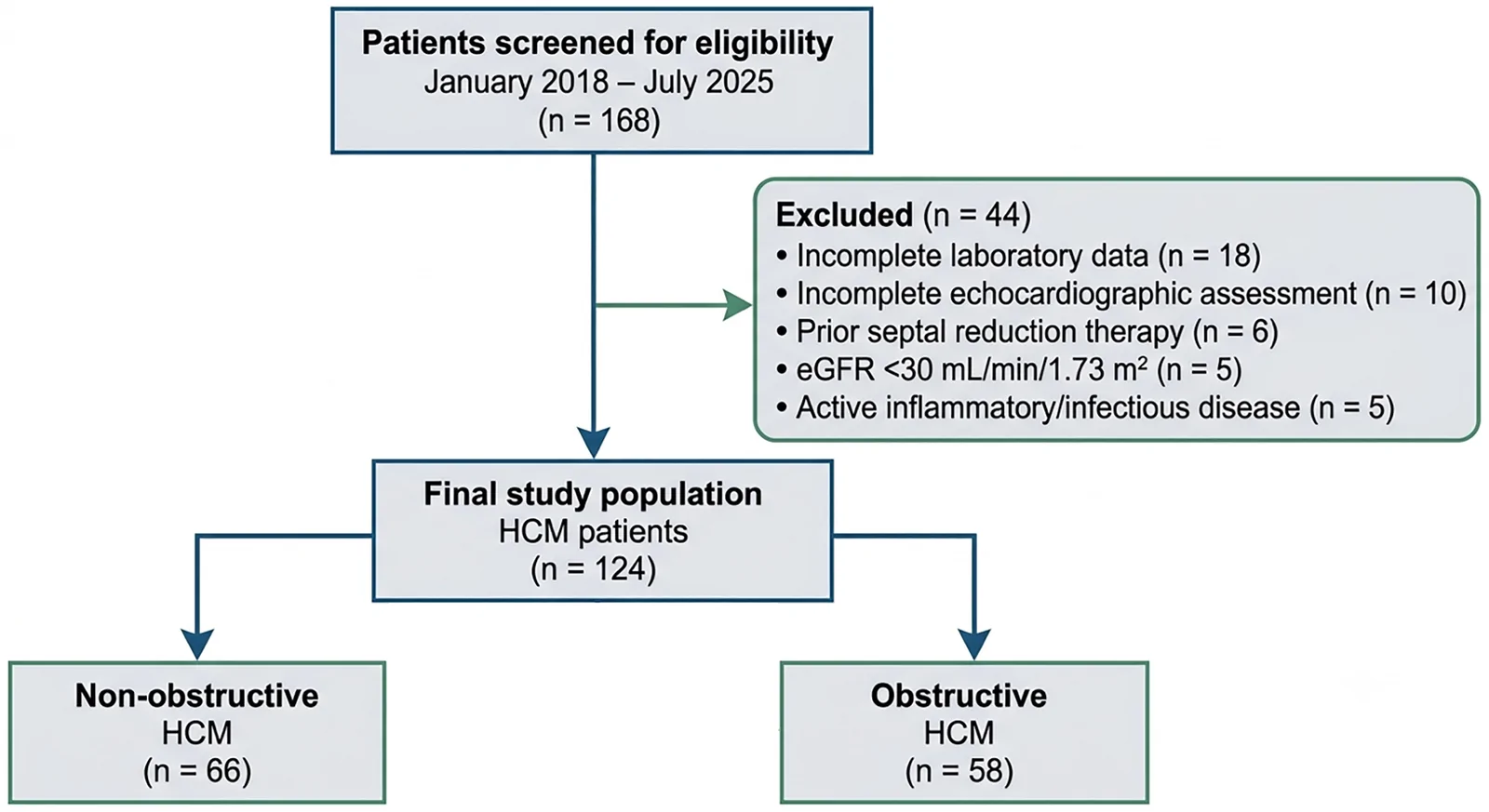
**Flowchart of patient screening, exclusions, and classification of the final HCM cohort into obstructive and non-obstructive groups according to resting or provoked LVOT obstruction.** **Abbreviations:** eGFR, estimated glomerular filtration rate; HCM, hypertrophic cardiomyopathy; LVOT, left ventricular outflow tract; n, number of patients.

The diagnosis of HCM was established according to current guideline recommendations, requiring a maximum left ventricular wall thickness ≥15 mm in the absence of other causes of hypertrophy. Potential alternative causes of LVH were systematically excluded through clinical evaluation, electrocardiography, echocardiography, and review of available imaging and medical records. Patients with severe aortic stenosis, infiltrative cardiomyopathies (including cardiac amyloidosis), Fabry disease, athlete’s heart, or hypertensive heart disease considered sufficient to explain the degree of hypertrophy were not included.

Demographic variables included age, sex, and body mass index (BMI). Comorbidities (hypertension, diabetes mellitus, dyslipidemia, arrhythmia, and family history of HCM) and medication use (angiotensin-converting enzyme [ACE] inhibitors/angiotensin receptor blockers, statins, fibrates, and oral antidiabetic drugs) were recorded.

The study cohort was derived from our institutional HCM registry, which has been utilized in previous investigations addressing different research questions.

### Laboratory measurements

Laboratory values included fasting plasma glucose (FPG), triglycerides (TG), C-reactive protein (CRP), and creatinine.

Blood samples were obtained after an overnight fast of at least 8–12 h. Fasting glucose and triglyceride measurements used for TyG calculation were obtained from the same blood sample. Laboratory measurements were performed within 30 days of the echocardiographic examination; when multiple measurements were available, the values closest to the echocardiographic assessment were used.

The TyG index was calculated according to the validated formula:

TyG index = ln [TG (mg/dL) × FPG (mg/dL) / 2]

The TyG index was calculated individually for each patient. Group values are presented as the mean of individual TyG measurements rather than values derived from group-level averages of triglycerides and fasting glucose.

### Echocardiographic assessment

Two-dimensional transthoracic echocardiography was performed using standard guideline-based protocols.

Measurements included interventricular septal thickness, posterior wall thickness, left atrial diameter, and left ventricular ejection fraction (LVEF).

LVOT gradient was assessed at rest and during provocation. Resting obstruction was defined as a peak LVOT gradient ≥30 mmHg at baseline echocardiography. In patients without resting obstruction, provocation testing using the Valsalva maneuver and/or exercise was performed when feasible. Obstructive HCM was defined as a peak LVOT gradient ≥30 mmHg at rest or during provocation. Of the 58 patients classified as having obstructive HCM, 52 had resting obstruction, and 6 were identified only during provocation, predominantly performed using the Valsalva maneuver, with exercise testing used in a limited number of cases based on local clinical practice.

The presence of systolic anterior motion (SAM) of the mitral valve and the degree of mitral regurgitation were recorded.

All echocardiographic examinations were interpreted by experienced cardiologists who were blinded to the TyG index values at the time of image analysis. Care was taken to align continuous-wave Doppler interrogation with LVOT flow and to minimize contamination from mitral regurgitation signals whenever possible.

### Ethical statement

The study was conducted in accordance with the Declaration of Helsinki and approved by the Institutional Ethics Committee of Akdeniz University (Approval number: TBAEK-435, approval date: 30 April 2026). Patient consent was waived due to the retrospective nature of the study and the use of anonymized clinical data.

### Statistical analysis

Continuous variables were expressed as mean ± standard deviation, and categorical variables as counts and percentages.

The distribution of continuous variables was assessed using visual inspection of histograms and the Shapiro–Wilk test. Normally distributed variables were compared using the independent-samples *t*-test, whereas non-normally distributed variables were evaluated using the Mann–Whitney *U* test. Categorical variables were compared using the chi-square test or Fisher’s exact test, as appropriate.

Pearson correlation analysis was initially used to examine associations between the TyG index and echocardiographic variables. Because LVOT gradients and inflammatory markers may exhibit non-normal distributions, additional sensitivity analyses were performed using Spearman rank correlation coefficients.

Binary logistic regression analysis was performed as an exploratory analysis to identify variables independently associated with LVOT obstruction. Covariates included age, sex, BMI, hypertension, diabetes mellitus, eGFR, CRP level, and septal thickness, based on clinical relevance and previously established associations with the HCM phenotype. Family history of HCM and medication use were not included in the primary model to avoid increasing model complexity relative to the number of obstructive events and mitigate the risk of model overfitting. Variables considered potential consequences of LVOT obstruction (e.g., SAM and mitral regurgitation) were not entered into the primary multivariable model to avoid overadjustment.

To explore whether the association between TyG and LVOT obstruction was driven primarily by triglycerides or fasting glucose, an additional sensitivity analysis was performed, replacing the TyG index with its individual components (triglycerides and FPG) in the multivariable model.

Odds ratios (ORs) with 95% confidence intervals (CIs) were reported. Multicollinearity among independent variables was assessed using variance inflation factor (VIF) analysis.

Receiver operating characteristic (ROC) curve analysis was conducted to evaluate the ability of the TyG index to discriminate between obstructive and non-obstructive HCM. The area under the curve (AUC), sensitivity, and specificity were calculated. The optimal cut-off value was determined using the maximum Youden index derived from ROC curve coordinates.

A two-sided *P*-value <0.05 was considered statistically significant. All analyses were performed using Statistical Package for the Social Sciences (SPSS) version 27 (IBM Corp., Armonk, NY, USA).

## Results

A total of 124 patients with HCM were enrolled, comprising 66 with non-obstructive and 58 with obstructive physiology. Patient selection is detailed in Figure 1, and baseline characteristics are summarized in Table 1.

**Table 1:** Baseline characteristics of patients with hypertrophic cardiomyopathy: Demographic, clinical, laboratory, and echocardiographic data

**Variables**	**Non-obstructive HCM (*n* ═ 66)**	**Obstructive HCM (*n* ═ 58)**	***P* value**
*Demographics*			
Age (years)	52.1 ± 5.8	51.2 ± 6.3	0.41
Male sex, *n* (%)	43 (65.2)	36 (62.1)	0.85
BMI (kg/m^2^)	25.9 ± 2.2	27.3 ± 2.3	0.001
Comorbidities			
Hypertension, *n* (%)	24 (36.4)	20 (34.5)	0.83
Diabetes mellitus, *n* (%)	15 (22.7)	13 (22.4)	0.967
Dyslipidemia, *n* (%)	6 (9.1)	7 (12.1)	0.77
Arrhythmia, *n* (%)	6 (9.1)	8 (13.8)	0.41
Family history of HCM, *n* (%)	22 (33.3)	31 (53.4)	0.03
*Laboratory values*			
FPG (mg/dL)	100.5 ± 17.4	102.3 ± 17.0	0.57
Triglycerides (mg/dL)	145.7 ± 19.9	173.2 ± 17.7	<0.001
TyG index	8.76 ± 0.21	9.07 ± 0.25	<0.001
CRP (mg/L)	4 (3–6)	8 (6–9)	<0.001
Creatinine (mg/dL)	0.93 ± 0.15	0.95 ± 0.18	0.53
eGFR (mL/min/1.73 m^2^)	77.9 ±13.8	80.5 ± 14.86	0.318
*Echocardiography*			
Septal thickness (mm)	17.5 ± 1.7	18.9 ± 2.1	<0.001
Posterior wall thickness (mm)	13.4 ± 1.4	14.1 ± 1.5	0.011
LVOT gradient (rest, mmHg)	17.1 ± 4.7	54.9 ± 10.7	<0.001
LVOT gradient (provocation, mmHg)	22.9 ± 4.6	69.1 ± 11.7	<0.001
Left atrial diameter (mm)	35.3 ± 3.5	38.6 ± 4.2	<0.001
LVEF (%)	65.1 ± 3.4	64.6 ± 3.6	0.44
SAM, *n* (%)	17 (25.8)	36 (62.1)	<0.001
Mitral regurgitation ≥ moderate, *n* (%)	7 (10.6)	15 (25.9)	0.03

Data are presented as mean ± standard deviation, median (interquartile range), or number (percentage), as appropriate. Group comparisons were performed using independent samples *t*-tests or Mann–Whitney *U* tests for continuous variables, and chi-square tests or Fisher’s exact tests for categorical variables. A *P* value of <0.05 was considered statistically significant. **Abbreviations**: BMI, body mass index; CRP, C-reactive protein; eGFR, estimated glomerular filtration rate; FPG, fasting plasma glucose; HCM, hypertrophic cardiomyopathy; LVEF, left ventricular ejection fraction; LVOT, left ventricular outflow tract; SAM, systolic anterior motion; TyG, triglyceride–glucose.

Age and sex distributions were comparable between the groups. Patients with LVOT obstruction exhibited a higher BMI (27.3 ± 2.3 vs. 25.9 ± 2.2 kg/m^2^, *P ═* 0.001) and a greater prevalence of positive family history (53.4% vs. 33.3%, *P ═* 0.03), while other clinical comorbidities were similar.

Patients with LVOT obstruction demonstrated elevated triglyceride levels (173.2 ± 17.7 vs. 145.7 ± 19.9 mg/dL, *P <* 0.001), higher TyG index values (9.07 ± 0.25 vs. 8.76 ± 0.21, *P <* 0.001), and CRP levels [median 8 (IQR 6–9) vs. 4 (IQR 3–6), *P <* 0.001]. Fasting glucose and creatinine levels did not significantly differ between groups.

Echocardiographic assessments revealed greater septal thickness (18.9 ± 2.1 vs. 17.5 ± 1.7 mm, *P <* 0.001), posterior wall thickness (14.1 ± 1.5 vs. 13.4 ± 1.4 mm, *P ═* 0.011), and left atrial diameter (38.6 ± 4.2 vs. 35.3 ± 3.5 mm, *P <* 0.001) in patients with obstruction. LVOT gradients were significantly higher both at rest (54.9 ± 10.7 vs. 17.1 ± 4.7 mmHg, *P <* 0.001) and with provocation (69.1 ± 11.7 vs. 22.9 ± 4.6 mmHg, *P <* 0.001). Systolic anterior motion (62.1% vs. 25.8%, *P <* 0.001) and at least moderate mitral regurgitation (25.9% vs. 10.6%, *P ═* 0.03) were more prevalent in the obstructive group, whereas LVEF was preserved and similar across both groups.

Medication usage did not vary significantly between the groups (Table S1).

In multivariable logistic regression analysis, the TyG index remained independently associated with LVOT obstruction. Each 0.1-unit increase in the TyG index correlated with a higher odds of obstruction (OR: 1.84, 95% CI: 1.40–2.42, *P <* 0.001). CRP was also independently associated with LVOT obstruction (OR: 1.54, 95% CI: 1.25–1.90, *P <* 0.001), while other variables lacked statistical significance. Model calibration was adequate, as indicated by the Hosmer–Lemeshow goodness-of-fit test (χ^2^ = 7.719, *P ═* 0.461), and the model explained a substantial proportion of the variance (Nagelkerke R^2^ = 0.643) (Table 2).

**Table 2:** Multivariable logistic regression analysis of variables independently associated with LVOT obstruction in patients with hypertrophic cardiomyopathy

**Variable**	**B**	***P* value**	**OR**	**95% CI (OR)**
Age (years)	−0.038	0.425	0.963	0.877–1.057
Male sex	−0.276	0.713	0.759	0.174–3.304
BMI (kg/m^2^)	0.217	0.076	1.242	0.978–1.578
Hypertension	−0.689	0.229	0.502	0.163–1.544
Diabetes mellitus	−0.230	0.733	0.795	0.213–2.969
Septal thickness (mm)	0.232	0.052	1.261	0.998–1.594
CRP (mg/L)	0.431	<0.001	1.539	1.249–1.898
TyG index (per 0.1 unit)	0.609	<0.001	1.838	1.399–2.415
eGFR (mL/min/1.73 m^2^)	0.014	0.592	1.014	0.964–1.066

ORs are presented per unit increase unless otherwise specified. The TyG index was scaled per 0.1-unit increment. **Abbreviations**: B, regression coefficient; BMI, body mass index; CI, confidence interval; CRP, C-reactive protein; eGFR, estimated glomerular filtration rate; HCM, hypertrophic cardiomyopathy; LVOT, left ventricular outflow tract; OR, odds ratio; TyG, triglyceride–glucose.

**Figure 2.**
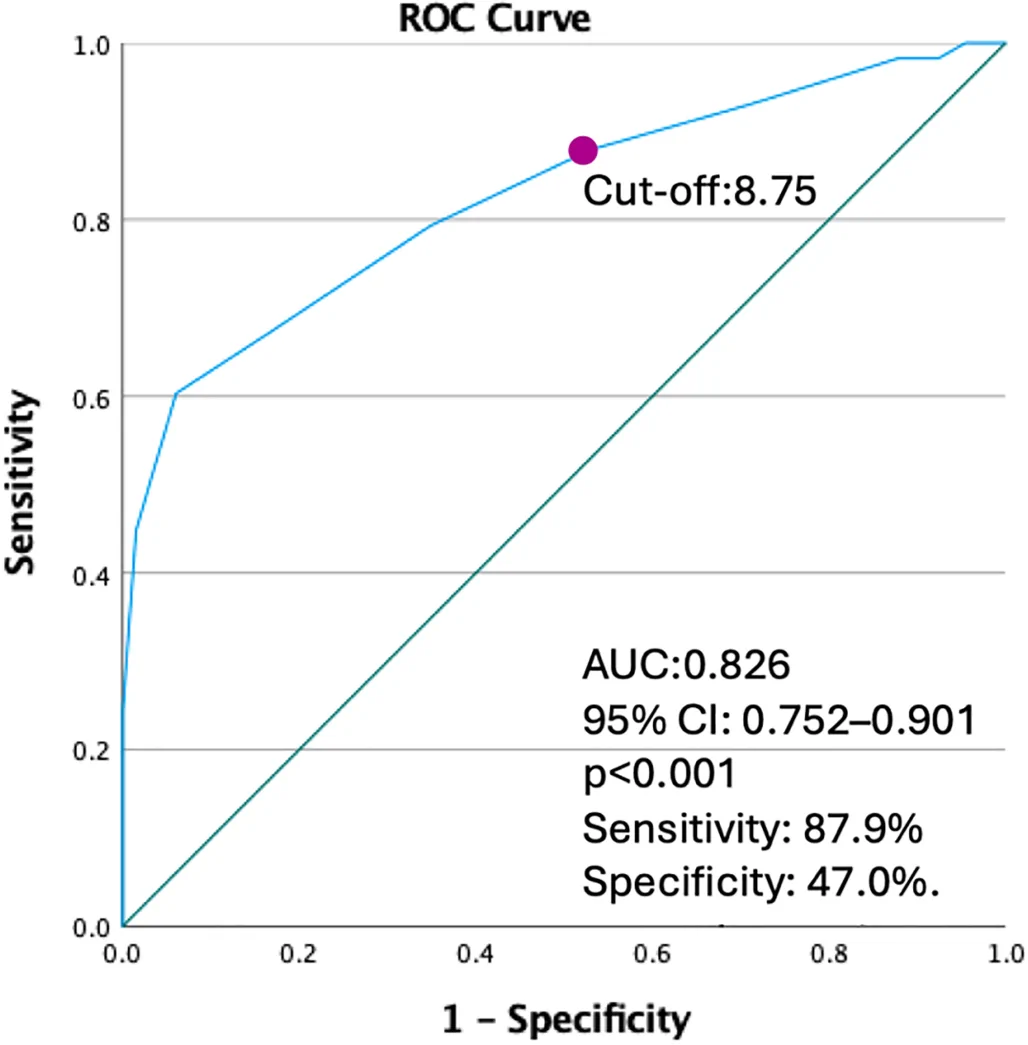
**Receiver operating characteristic curve of the TyG index for identifying LVOT obstruction in patients with HCM.** ROC curve analysis showed good discriminatory performance of the TyG index for distinguishing obstructive from non-obstructive HCM, with an AUC of 0.826 (95% CI: 0.752–0.901; *P <* 0.001). The optimal cut-off value, determined using the maximum Youden index, was 8.75, yielding a sensitivity of 87.9% and a specificity of 47.0%. The diagonal reference line represents no discriminatory ability. Given the modest specificity, this threshold should be interpreted as exploratory rather than diagnostic. **Abbreviations**: AUC, area under the curve; CI, confidence interval; HCM, hypertrophic cardiomyopathy; LVOT, left ventricular outflow tract; ROC, receiver operating characteristic; TyG, triglyceride–glucose.

The TyG index demonstrated a moderate positive correlation with LVOT gradient at rest (*r* ═ 0.519, *P <* 0.001) and with provocation (*r* ═ 0.557, *P <* 0.001). No significant correlations were observed with septal thickness, left atrial diameter, or LVEF.

Sensitivity analyses using Spearman rank correlation confirmed the robust association between the TyG index and LVOT gradients, both at rest (ρ = 0.494, *P <* 0.001) and during provocation (ρ = 0.535, *P <* 0.001).

To further investigate the individual contributions of the TyG index components, an additional multivariable logistic regression model was constructed using FPG and triglyceride levels. In this model, triglyceride level remained independently associated with LVOT obstruction (OR: 1.082 per mg/dL increase, *P <* 0.001), whereas FPG was not significantly associated (OR: 1.012, *P ═* 0.484). CRP and septal thickness also remained independently associated with LVOT obstruction in this analysis.

ROC curve analysis revealed that the TyG index exhibited good discriminatory performance for LVOT obstruction, with an AUC of 0.826 (95% CI: 0.752–0.901, *P <* 0.001) (Figure 2). The optimal cut-off value, determined by the maximum Youden index, was 8.75 (Youden index, 0.35), yielding a sensitivity of 87.9% and a specificity of 47.0%.

## Discussion

In this retrospective observational study, we demonstrated that the TyG index was significantly elevated in patients with LVOT obstruction and remained independently associated with obstruction after adjustment for relevant clinical and echocardiographic variables. This association, consistent when the TyG index was modeled per 0.1-unit increment, supports the robustness and interpretability of our findings.

Furthermore, the association between the TyG index and LVOT gradients remained significant in sensitivity analyses using Spearman rank correlations, indicating that the observed relationship was not solely dependent on assumptions of normal data distribution.

HCM is characterized by substantial phenotypic heterogeneity, which cannot be fully explained by sarcomeric mutations alone. Recent evidence highlights the contribution of both sarcomeric and non-sarcomeric genetic variants [12], underscoring the role of non-genetic modifiers in disease expression. In this context, metabolic dysregulation may represent a critical factor influencing myocardial remodeling and the development of obstructive physiology.

Insulin resistance has been linked to impaired myocardial energy utilization, characterized by reduced glucose uptake and increased reliance on lipid metabolism, potentially promoting hypertrophic remodeling [13]. Additionally, endothelial dysfunction and microvascular impairment, common features of insulin-resistant states, may contribute to altered myocardial perfusion and increased hemodynamic burden [14]. While these mechanisms offer biologically plausible links between metabolic abnormalities and HCM phenotypes, the retrospective cross-sectional design of the present study precludes establishing a causal relationship between metabolic dysregulation and LVOT obstruction.

Beyond insulin resistance, alterations in myocardial energetics may also contribute to phenotypic variability in HCM. Previous experimental and clinical studies have demonstrated impaired energy utilization and abnormal substrate metabolism in hypertrophied myocardium [15, 16]. Increased reliance on fatty acid metabolism coupled with reduced glucose utilization may further impair myocardial efficiency and promote maladaptive remodeling. Although a detailed mechanistic evaluation was beyond the scope of this study, these observations may partly elucidate the relationship between metabolic status and LVOT obstruction observed in our cohort.

The TyG index, a composite marker derived from fasting glucose and triglyceride levels, offers a practical estimate of insulin resistance. Prior studies have demonstrated associations between elevated TyG and LVH, diastolic dysfunction, and adverse cardiovascular outcomes across diverse populations [17–20]. In coronary artery disease, higher TyG levels have been associated with impaired myocardial function [21], and an increased risk of atrial fibrillation has also been reported [22]. However, data specific to HCM remain limited and heterogeneous.

Prior studies on the TyG index within HCM populations have yielded inconsistent findings. For instance, Wei et al. demonstrated that TyG independently predicted postoperative atrial fibrillation following septal myectomy [23], while Meng et al. reported an inverse association between TyG and obstructive HCM in non-diabetic individuals [24]. Conversely, Liu et al. highlighted its prognostic relevance in HCM patients with heart failure with preserved ejection fraction [25]. These discrepancies may stem from variations in study design, metabolic profiles, inclusion criteria, and patient selection. Our research expands upon this evidence by revealing a positive association between TyG and LVOT obstruction in a broader HCM cohort.

Crucially, the observed relationship in our study appears to be predominantly driven by triglyceride levels rather than fasting glucose. In a multivariable model incorporating individual components of the TyG index, triglyceride levels maintained an independent association with LVOT obstruction, whereas FPG did not. This suggests that lipid-related metabolic abnormalities may contribute more substantially to the identified association within our cohort. Consequently, these findings should not be interpreted as direct evidence of heightened insulin resistance in patients with obstructive HCM. Direct measures of insulin resistance, such as fasting insulin levels, homeostatic model assessment of insulin resistance (HOMA-IR), or hyperinsulinemic-euglycemic clamp testing, were unavailable for this analysis.

Emerging evidence also posits that metabolic dysfunction-associated steatotic liver disease (MASLD) may represent another manifestation of the systemic metabolic milieu linked to adverse cardiovascular remodeling [26]. Shared mechanisms, including insulin resistance, chronic low-grade inflammation, oxidative stress, and lipid dysregulation, have been implicated in both MASLD and myocardial structural abnormalities. Although liver-related parameters were not assessed in the current study, elevated TyG values may reflect a broader cardiometabolic phenotype extending beyond glucose metabolism alone.

Beyond the TyG index, nascent data support the role of other metabolic-inflammatory markers in HCM. Notably, the uric acid-to-albumin ratio has been associated with adverse clinical outcomes in HCM patients [27], reinforcing the concept that systemic metabolic and inflammatory pathways may contribute to disease expression and progression. Collectively, these observations suggest that a multidimensional metabolic profile may be pertinent to understanding phenotypic variability in HCM.

While ROC analysis demonstrated good overall discrimination (AUC 0.826), the optimal cut-off value exhibited relatively low specificity. Therefore, the TyG index should not be considered a diagnostic or screening tool for LVOT obstruction. Instead, it may serve as a supportive marker reflecting underlying metabolic status. Furthermore, as the cut-off value was derived and evaluated within the same cohort, external validation is imperative before considering any clinical application.

From a clinical standpoint, the TyG index is cost-effective, widely accessible, and easily derived from routine laboratory tests. These attributes make it a practical parameter for clinical research and exploratory phenotyping studies. However, the present study did not evaluate whether the TyG index provides incremental information beyond established echocardiographic or clinical predictors, thus leaving its added clinical utility uncertain.

Several limitations warrant acknowledgment. First, the retrospective observational design restricts causal inference and may introduce selection bias and residual confounding. Second, the sample size was relatively modest, and external validation was not performed. Additionally, the number of obstructive cases was limited relative to the covariates included in the multivariable model, raising the possibility of model overfitting; therefore, the adjusted estimates should be considered exploratory. Third, direct measurements of insulin resistance, including fasting insulin, HOMA-IR, and HbA1c, were not assessed. Fourth, advanced imaging parameters such as myocardial strain analysis or cardiac magnetic resonance-derived fibrosis assessment were unavailable. Important HCM-related characteristics, including genotype status, septal morphology, mitral valve anatomical features, New York Heart Association (NYHA) functional class, and myocardial fibrosis burden, were not systematically available and thus could not be incorporated into the analyses.

LVOT obstruction was defined using both resting and provoked gradients. However, provocation testing was not fully standardized across all patients, as exercise-based provocation was not feasible in every case, and medication status was not uniformly controlled before testing. Consequently, some degree of classification bias cannot be excluded.

Finally, although the TyG index demonstrated good discriminatory performance, the identified cut-off value was derived and evaluated within the same study cohort and was not internally or externally validated. Accordingly, this threshold should be considered exploratory and interpreted with caution until confirmed in independent prospective studies.

Future prospective multicenter studies are essential to validate these findings and to elucidate the clinical significance of metabolic markers in HCM. Particular attention should be directed toward determining whether TyG provides prognostic information beyond conventional echocardiographic assessment and whether metabolic interventions influence disease expression or progression.

## Conclusion

The TyG index was independently associated with LVOT obstruction in patients with HCM. Higher TyG values were observed in patients with obstructive physiology and correlated with both resting and provoked LVOT gradients. These findings suggest that metabolic abnormalities may be associated with phenotypic variation in HCM. However, given the retrospective observational design, the limited sample size, and the absence of direct measurements of insulin resistance, the results should be interpreted as an association rather than evidence of a causal relationship. The TyG index may represent a readily obtainable marker of metabolic status in patients with HCM, but its clinical utility and incremental value beyond established clinical and echocardiographic parameters remain uncertain. Further prospective multicenter studies are required to validate these findings and determine their potential clinical significance.

## Supplemental data

**Table S1:** Medication use in obstructive versus non-obstructive HCM cohorts

**Medication class**	**Non-obstructive (*n* ═ 66)**	**Obstructive (*n* ═ 58)**	***P* value**
Statins, n (%)	6 (9.1%)	7 (12.1%)	0.77
Fibrates, n (%)	4 (6.1%)	4 (6.9%)	0.817
Oral antidiabetic drugs, n (%)	15 (22.7%)	13 (22.4%)	0.967
Beta-blockers, n (%)	57 (86.4%)	53 (91.4%)	0.41
ACEi/ARB, n (%)	11 (16.7%)	10 (17.2%)	0.958

Data are presented as *n* (%). Categorical variables were compared using the Pearson chi-square test or Fisher’s exact test, as appropriate, based on expected cell counts. **Abbreviations**: ACEi/ARB, angiotensin-converting enzyme inhibitor/angiotensin receptor blocker; HCM, hypertrophic cardiomyopathy.

## Data Availability

The data underlying this article will be shared by the corresponding author upon reasonable request.

## References

[1] Maron BJ, Gardin JM, Flack JM, Gidding SS, Kurosaki TT, Bild DE (1995). Prevalence of hypertrophic cardiomyopathy in a general population of young adults: echocardiographic analysis of 4111 subjects in the CARDIA Study. Circulation.

[2] Arbelo E, Protonotarios A, Gimeno JR, Arbustini E, Barriales-Villa R, Basso C (2023). 2023 ESC guidelines for the management of cardiomyopathies. Eur Heart J.

[3] Maron MS, Olivotto I, Betocchi S, Casey SA, Lesser JR, Losi MA (2003). Effect of left ventricular outflow tract obstruction on clinical outcome in hypertrophic cardiomyopathy. N Engl J Med.

[4] Geske JB, Ommen SR, Gersh BJ (2018). Hypertrophic cardiomyopathy: clinical update. JACC Heart Fail.

[5] Ommen SR, Mital S, Burke MA, Day SM, Deswal A, Elliott P (2020). 2020 AHA/ACC guideline for the diagnosis and treatment of patients with hypertrophic cardiomyopathy: a report of the American College of Cardiology/American Heart Association Joint Committee on Clinical Practice Guidelines. J Am Coll Cardiol.

[6] Reaven GM (2011). Insulin resistance: the link between obesity and cardiovascular disease. Med Clin North Am.

[7] Simental-Mendía LE, Rodríguez-Morán M, Guerrero-Romero F (2008). The product of fasting glucose and triglycerides as surrogate for identifying insulin resistance in apparently healthy subjects. Metab Syndr Relat Disord.

[8] Sánchez-Íñigo L, Navarro-González D, Fernández-Montero A, Pastrana-Delgado J, Martínez JA (2016). The TyG index may predict the development of cardiovascular events. Eur J Clin Invest.

[9] Won KB, Park EJ, Han D, Lee JH, Choi SY, Chun EJ (2020). Triglyceride–glucose index is an independent predictor for the progression of coronary artery calcification in the absence of heavy coronary artery calcification at baseline. Cardiovasc Diabetol.

[10] Jv M, Zhang J, Han Y, Yang A, Zhu Y (2024). Association of triglyceride glucose-body mass index and left ventricular hypertrophy in patients with IgA nephropathy: a retrospective study. Eur J Med Res.

[11] Yang C, Liu W, Tong Z, Lei F, Lin L, Huang X (2024). The relationship between insulin resistance indicated by triglyceride–glucose index and left ventricular hypertrophy and decreased left ventricular diastolic function with preserved ejection fraction. Diabetes Metab Syndr Obes.

[12] Oktay V, Tüfekçioğlu O, Yılmaz DÇ, Onrat E, Karabulut D, Çelik M (2023). The definition of sarcomeric and non-sarcomeric gene mutations in hypertrophic cardiomyopathy patients: a multicenter diagnostic study across Türkiye. Anatol J Cardiol.

[13] Jia G, Hill MA, Sowers JR (2018). Diabetic cardiomyopathy: an update of mechanisms contributing to this clinical entity. Circ Res.

[14] Bonetti PO, Lerman LO, Lerman A (2003). Endothelial dysfunction: a marker of atherosclerotic risk. Arterioscler Thromb Vasc Biol.

[15] Bayraktar A, Canpolat U, Demiri E, Kunak AU, Ozer N, Aksoyek S (2015). New insights into the mechanisms of diastolic dysfunction in patients with type 2 diabetes. Scand Cardiovasc J.

[16] Crilley JG, Boehm EA, Blair E, Rajagopalan B, Blamire AM, Styles P (2003). Hypertrophic cardiomyopathy due to sarcomeric gene mutations is characterized by impaired energy metabolism irrespective of the degree of hypertrophy. J Am Coll Cardiol.

[17] Cetin Sanlialp S, Sanlialp M, Nar G, Malcok A (2022). Triglyceride–glucose index reflects the unfavorable changes of left ventricular diastolic functions and structure in uncomplicated newly diagnosed hypertensive patients. Clin Exp Hypertens.

[18] Sun QC, Liu J, Meng R, Zhang N, Yao J, Yang F (2023). Association of the triglyceride–glucose index with subclinical left ventricular dysfunction in type 2 diabetes mellitus patients: a retrospective cross-sectional study. J Diabetes Investig.

[19] Chen Y, Fu J, Wang Y, Zhang Y, Shi M, Wang C (2023). Association between triglyceride–glucose index and subclinical left ventricular systolic dysfunction in patients with type 2 diabetes. Lipids Health Dis.

[20] Khalaji A, Behnoush AH, Khanmohammadi S, Ghanbari Mardasi K, Sharifkashani S, Sahebkar A (2023). Triglyceride–glucose index and heart failure: a systematic review and meta-analysis. Cardiovasc Diabetol.

[21] Na L, Cui W, Li X, Chang J, Xue X (2023). Association between the triglyceride–glucose index and left ventricular global longitudinal strain in patients with coronary heart disease in Jilin Province, China: a cross-sectional study. Cardiovasc Diabetol.

[22] Shi S, Song Y, Liu Z, He J, Zheng Z, Song C (2025). The association of the triglyceride–glucose index with the risk of atrial fibrillation: analysis of the UK Biobank. Nutr Metab Cardiovasc Dis.

[23] Wei Z, Zhu E, Ren C, Dai J, Li J, Lai Y (2021). Triglyceride–glucose index independently predicts new-onset atrial fibrillation after septal myectomy for hypertrophic obstructive cardiomyopathy beyond the traditional risk factors. Front Cardiovasc Med.

[24] Meng X, Gao J, Zhang K, Jun W, Wang JJ, Wang XL (2023). The triglyceride–glucose index as a potential protective factor for hypertrophic obstructive cardiomyopathy without diabetes: evidence from a two-center study. Diabetol Metab Syndr.

[25] Liu L, Zheng Y, Ma H, Liao Q, Liu MJ, Ren H (2025). Association between triglyceride–glucose index and prognosis of patients with hypertrophic cardiomyopathy and heart failure with preserved ejection fraction. Anatol J Cardiol.

[26] Sonaglioni A, Cerini F, Fagiani V, Nicolosi GL, Rumi MG, Lombardo M (2025). Effect of metabolic dysfunction-associated steatotic liver disease on left ventricular mechanics in patients without overt cardiac disease: a systematic review and meta-analysis. J Clin Med.

[27] Ülgen Kunak A, Kunak T (2026). Association between uric acid-to-albumin ratio and clinical outcomes in patients with hypertrophic cardiomyopathy: a retrospective observational study. J Int Med Res.

